# Nondestructive Detection of Eggshell Thickness Using Near-Infrared Spectroscopy Based on GBDT Feature Selection and an Improved CatBoost Algorithm

**DOI:** 10.3390/foods15081286

**Published:** 2026-04-08

**Authors:** Ziqing Li, Ying Ji, Changheng Zhao, Dehe Wang, Rongyan Zhou

**Affiliations:** 1 College of Information Science and Technology, Hebei Agricultural University, Baoding 071001, China; lzqingwork@163.com; 2 Hebei Key Laboratory of Agricultural Big Data, Baoding 071001, China; 3 College of Animal Science and Technology, Hebei Agricultural University, Baoding 071001, China; chzsdau@163.com (C.Z.); theconcertevent@163.com (D.W.); zhry@hebau.edu.cn (R.Z.)

**Keywords:** eggshell thickness, spectral analysis, GBDT, CatBoost, feature optimization, non-destructive testing

## Abstract

Eggshell thickness is a critical indicator for evaluating egg breakage resistance and hatchability, yet traditional measurement methods remain destructive and inefficient. To address this, this study proposes a robust prediction approach by integrating Gradient Boosting Decision Tree (GBDT) feature optimization with an improved CatBoost algorithm. First, a joint strategy of Standard Normal Variate (SNV) and Multiplicative Scatter Correction (MSC) was employed to eliminate spectral scattering noise and enhance organic matrix fingerprint information. Subsequently, GBDT was introduced for nonlinear feature evaluation to adaptively screen the top 50 wavelengths, effectively mitigating the “curse of dimensionality” and multicollinearity in full-spectrum data. A CatBoost regression model was then constructed using an Ordered Boosting mechanism, supported by a dual anti-overfitting strategy that merged 10-fold nested cross-validation with Bootstrap resampling. Experimental results demonstrate that this method significantly outperforms traditional algorithms in both prediction accuracy and generalization. The coefficients of determination (R^2^) for the calibration and prediction sets reached 0.930 and 0.918, respectively, with a root mean square error of prediction (RMSEP) of 0.008 mm. Residual analysis confirms that prediction errors follow a zero-mean Gaussian distribution, indicating that systematic bias was effectively eliminated. This research provides a reliable theoretical foundation and technical support for the intelligent grading of poultry egg quality.

## 1. Introduction

Eggs represent a globally consumed, premium source of animal protein. Maintaining their structural integrity during harvesting, automated grading, packaging, and long-distance transport is critical to safeguarding the economic viability of the poultry industry [1]. From a food science and safety perspective, the eggshell acts as the primary physical barrier against foodborne pathogens; thus, its thickness directly dictates the consumption safety and internal quality stability of eggs throughout distribution and storage [2]. Furthermore, as both a protective shield and a vital calcium reservoir for embryonic development, the microstructural and macroscopic dimensions of the eggshell fundamentally govern the impact resistance, storage durability, and hatchability of the eggs [3,4]. Current statistics reveal that shell defects—primarily inadequate or uneven thickness—result in a 5% to 8% breakage rate during commercial circulation. This vulnerability not only triggers profound direct economic losses but also severely exacerbates the risk of pathogenic invasion and cross-contamination by bacteria such as Salmonella, thereby posing a significant threat to public health [5,6]. Consequently, developing rapid, accurate, and non-destructive detection technologies to supersede inefficient conventional spot-checking has become an urgent prerequisite for the modern food industry to implement comprehensive online quality control and intelligent grading [7,8].

At present, the standard benchmark for eggshell thickness measurement is predominantly reliant on contact-based instruments, such as digital micrometers or vernier calipers. While these techniques provide accurate numerical data, their inherently “destructive” nature results in irreversible sample loss and limited throughput efficiency, failing to satisfy the high-volume processing requirements of tens of thousands of units per hour in modern industrial production lines [9]. To address these constraints, researchers have explored various NDT technologies. Kibala et al. [10] investigated the application of ultrasonic technology in eggshell thickness determination, confirming a high correlation with actual measurements; however, this approach typically necessitates coupling agents and is highly sensitive to the sensor’s contact angle, which limits its adaptability to high-speed, dynamic detection environments. Although machine vision can effectively extract external morphological features such as the egg shape index, it lacks the penetrative capability required to characterize internal structural information like thickness [11].

In contrast, near-infrared spectroscopy (NIRS) has emerged as a mainstream modality for the internal quality evaluation of agricultural products, owing to its advantages of being non-contact, non-polluting, and capable of instantaneous multi-component detection [12,13]. Despite the absence of direct absorption peaks for calcium carbonate (CaCO_3_)—the primary constituent of eggshells—in the near-infrared region, recent research by Ahmed et al. [14] indicates that NIRS can sensitively capture the overtone and combination vibrational information of hydrogen-containing groups (C-H, N-H, O-H) within the organic matrix (e.g., glycoproteins, membrane lipids, and moisture). Given that this organic matrix regulates the biomineralization process of the eggshell, a strong intrinsic mapping mechanism exists between the spectral response characteristics and the shell’s microscopic crystal structure and macroscopic thickness [15,16].

However, near-infrared spectral data are typically characterized by high dimensionality, nonlinearity, and strong multicollinearity. The vast amount of redundant information and background noise inherent in full-spectrum data often masks critical “thickness-related fingerprints.” While traditional Partial Least Squares Regression (PLSR) is robust in handling multicollinearity, it struggles to resolve the complex nonlinear dependencies between spectral responses and eggshell thickness. Conventional machine learning algorithms, such as Random Forest (RF) and Support Vector Machines (SVM), are frequently susceptible to interference from irrelevant variables when processing small-sample, high-dimensional spectral data. To mitigate feature redundancy, methods such as Competitive Adaptive Reweighted Sampling (CARS) have been widely adopted; nonetheless, these linear feature selection approaches tend to ignore the nonlinear interactions between variables [17,18]. Recently, GBDT has gained significant attention due to its powerful feature mining capabilities. However, standard GBDT algorithms exhibit “prediction shift” when handling numerical features and are prone to overfitting on training sets. This results in a significant discrepancy where calibration accuracy markedly exceeds prediction accuracy, thereby limiting the model’s generalization performance.

In view of these considerations, this study proposes a novel framework for the nondestructive detection of eggshell thickness, integrating gradient boosting feature optimization with an improved CatBoost algorithm. First, the feature importance evaluation mechanism of GBDT is utilized to adaptively select the top 50 feature wavelengths from the full spectra preprocessed by SNV and MSC, effectively mitigating the “curse of dimensionality.”

Second, the CatBoost algorithm, characterized by its Ordered Boosting mechanism, is introduced to construct the regression model. This approach effectively overcomes the gradient estimation bias inherent in traditional boosting methods, thereby enhancing the model’s prediction accuracy for unknown samples.

Finally, an anti-overfitting parameter optimization strategy based on 10-fold nested cross-validation is proposed. This strategy innovatively incorporates a Bootstrap resampling mechanism, employing random resampling techniques based on the Bernoulli distribution to increase the diversity of training samples. By synergistically constraining the learning rate and the L2 regularization coefficient, the proposed framework aims to achieve high-precision nondestructive detection of eggshell thickness using destructive measurements as the ground-truth benchmark. This research provides a solid theoretical foundation and technical support for the intelligent grading of egg products in modern production lines.

## 2. Materials and Methods

### 2.1. Materials and Data Acquisition

#### 2.1.1. Sample Preparation

A total of 500 fresh commercial brown-shelled eggs were selected as experimental samples, all of which were produced by Hebei Qianxiyuan Agricultural Development Co., Ltd. (Cangzhou, China). The samples originated from a core flock of laying hens during their peak laying period (200–300 days of age), with individual egg weights controlled within the range of 50–60 g. Given that laying hens at this stage exhibit vigorous metabolic activity and a stable eggshell calcification process, the selected samples are highly representative of the population in terms of physical and mechanical properties.

To eliminate interferences from surface contaminants and environmental temperature/humidity fluctuations during spectral collection, all eggs were gently wiped with distilled water to remove surface stains and subsequently air-dried naturally. Afterward, the samples were placed in a constant-temperature laboratory environment at 24 °C for 24 h to reach thermal equilibrium. Three points were randomly marked on the equatorial region of each egg to serve as corresponding sites for both spectral acquisition and thickness measurement. Extreme care was taken during the marking process to avoid any structural damage to the eggshell surface.

#### 2.1.2. Spectral Data Acquisition

**Figure 1:**
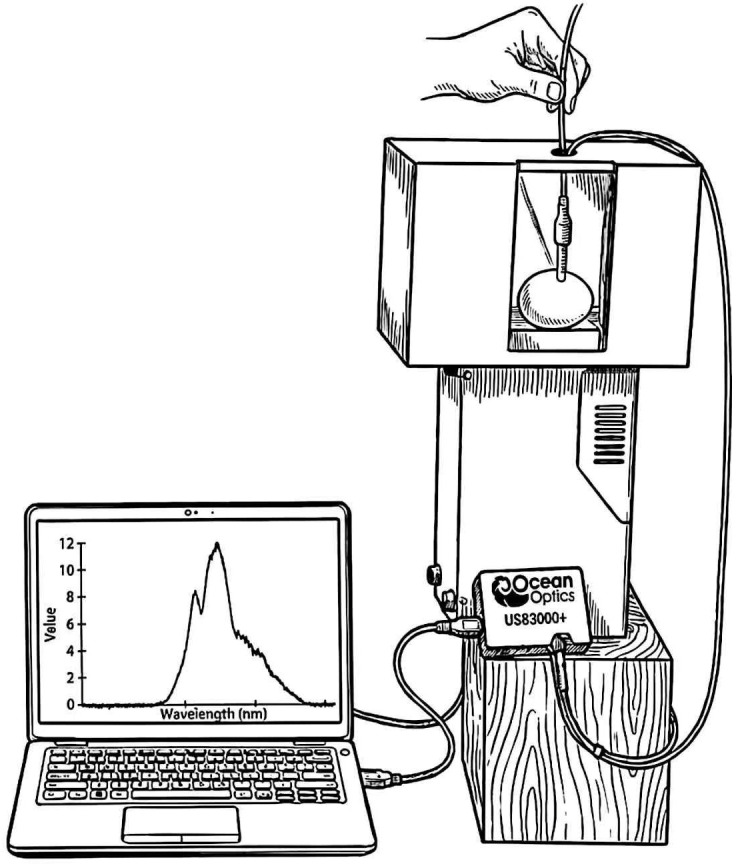
Spectral acquisition system.

Spectral data acquisition was performed using an Ocean Optics USB2000+ spectrometer (Ocean Optics, Orlando, FL, USA). The instrument operates within a wavelength range of 340–1032 nm, covering both the visible and short-wave near-infrared (SW-NIR) regions. The spectral acquisition system primarily consisted of a high-stability tungsten halogen light source, an optical lens assembly, a fiber optic probe, a dark box (light-tight enclosure), and spectral analysis software (SpectraSuite version 2.0, Ocean Optics, Orlando, FL, USA; Python version 3.10, Python Software Foundation, Wilmington, DE, USA). The detailed configuration of the system is illustrated in Figure 1.

During the data acquisition process, each egg sample was securely positioned on the transmission detection stage within a light-tight dark box to ensure stable light output and effectively shield the system from external ambient stray light interference. Spectral data were collected in transmission mode, with the fiber optic probe vertically aligned with the marked points on the equatorial region of the eggshell. This setup captured the transmitted light signals that had penetrated both the eggshell and its internal contents. These signals, carrying rich characteristic information regarding the eggshell’s microstructure and its organic matrix, were transmitted via optical fiber to the spectrometer for spectral dispersion and processing.

Real-time monitoring of the spectral response curves and values was performed using the proprietary SpectraSuite software (version 2.0, Ocean Optics, Inc.), which also facilitated automated data naming and storage. The acquisition parameters were configured as follows: an integration time of 100 ms, a smoothing boxcar width of 5, and an average of 10 scans per acquisition. Each marked site was scanned three times, and the arithmetic mean of these repetitions was calculated to serve as the raw transmission spectral data for the respective sample.

#### 2.1.3. Measurement of Reference Eggshell Thickness Values

Immediately following the spectral acquisition, destructive thickness measurements were performed on the samples. The eggshells were broken along the marked points used for spectral collection, and the inner shell membranes were carefully removed. The actual thickness at each marked site was then measured using a high-precision digital micrometer. This instrument features a resolution of 0.001 mm and is equipped with a ratchet stop mechanism to ensure the application of constant contact pressure during measurement, thereby eliminating minor deformation errors caused by variations in manual pressure.

The arithmetic mean of the measurements from the three marked sites was calculated and used as the reference thickness value for each egg sample. Statistical analysis indicated that the eggshell thicknesses of the 500 samples ranged from 0.280 to 0.440 mm. The data exhibited the characteristics of a normal distribution, satisfying the requirements for sample diversity in the subsequent modeling process.

### 2.2. Workflow of the Nondestructive Algorithm for Eggshell Thickness Detection

**Figure 2:**
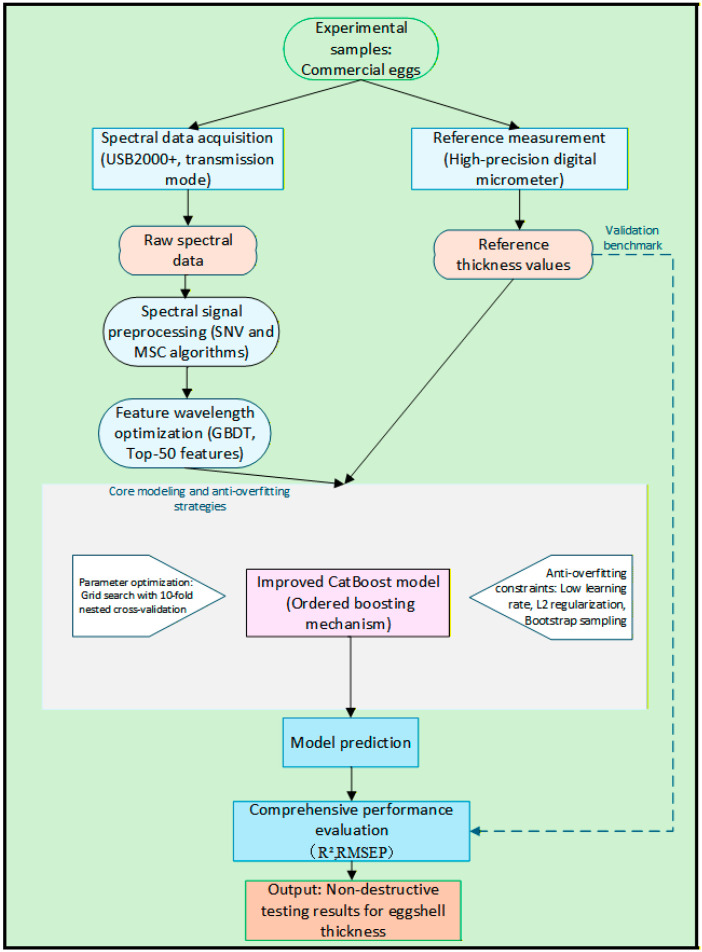
Flowchart of non-destructive eggshell thickness detection algorithm based on GBDT-CatBoost.

To ensure the robustness of detection accuracy and facilitate the in-depth extraction of thickness-related feature information within the spectra, a comprehensive technical roadmap was developed, encompassing signal purification, nonlinear feature optimization, and anti-overfitting modeling, as illustrated in Figure 2. The detection system initiates with spectral data preprocessing, where a joint SNV and MSC strategy is employed to effectively calibrate scattering errors arising from sample surface texture and optical path differences. This process eliminates physical noise and enhances the chemical fingerprint responses of the organic matrix. Building upon the high-quality preprocessed spectra, the workflow enters the critical feature extraction phase. A nonlinear mapping mechanism is established using the GBDT algorithm [19,20], through which the top 50 feature wavelengths with the highest contribution to the thickness response are adaptively selected based on the information gain criterion. This strategy successfully mitigates the “curse of dimensionality” associated with full-spectrum data while preserving essential core feature information.

In the subsequent model construction phase, an improved CatBoost algorithm [21,22,23,24], characterized by its Ordered Boosting mechanism, was integrated as the core regression engine. To specifically mitigate the high susceptibility of spectral data to overfitting, an innovative dual anti-overfitting framework was developed by combining 10-fold nested cross-validation with Bootstrap resampling. This workflow assesses generalization capability within the outer loops while identifying optimal hyperparameters through grid search within the inner loops. Furthermore, a stochastic sampling technique with a 0.8 sub-sampling rate was implemented to enhance the diversity of the training dataset, thereby compelling the model to capture globally robust features instead of memorizing local noise. Finally, the output results were systematically analyzed using performance metrics, including the R^2^ and RMSEP, to achieve precise evaluation and validation of eggshell thickness. This concludes the establishment of a complete technical scheme for real-time online detection.

### 2.3. Spectral Signal Preprocessing

Raw transmission spectral signals are inherently contaminated by high-frequency random interferences originating from light source instability, instrument thermal noise, and dark current. Furthermore, these signals are unavoidably coupled with baseline drift and multiple light scattering effects. Such physical interferences significantly obscure the subtle chemical fingerprints of the organic matrix associated with eggshell thickness, thereby directly undermining the precision and robustness of the subsequent predictive modeling.

To effectively mitigate background noise interference, calibrate optical path differences, and enhance the resolution of spectral features, this study comparatively investigated several widely used spectral preprocessing algorithms. The specific strategies included: the employment of Savitzky–Golay (SG) smoothing to suppress high-frequency random noise; the application of first-order (1st) and second-order (2nd) derivatives to eliminate baseline shifts and linear tilts; and the utilization of SNV and MSC to counteract multiplicative scattering errors resulting from the non-uniform distribution of sample particles. Among these techniques, SNV and MSC demonstrated particularly superior performance in calibrating light scattering effects.

#### 2.3.1. SNV

*SNV* is designed to standardize each individual spectrum, thereby eliminating spectral tilting and scaling effects caused by variations in sample surface texture and optical path length [25]. For the raw spectrum *x_i_* of the *i*-th sample, the correction formula is defined as:

(1) $$x_{i},SNV=\frac{x_{i}-\bar{x_{i}}}{\sigma_{i}}$$

where *x_i_*, *SNV* denotes the corrected spectral vector, $\bar{x_{i}}$ represents the mean absorbance across all wavelength points for that specific spectrum, and $\sigma_{i}$ is the standard deviation of the corresponding spectrum.

#### 2.3.2. MSC

*MSC* is designed to isolate and eliminate additive baseline drift and multiplicative signal distortion arising from non-uniform particle distribution and variations in scattering levels [26,27]. This is achieved by performing a linear regression of each individual sample spectrum against an ideal reference spectrum, which is typically defined as the mean spectrum of the entire dataset.

Assuming the mean spectrum is represented by $\bar{x}$, the regression model and the subsequent correction procedure for the *i*-th spectrum are expressed as:

(2) $$\begin{array}{l} x_{i}=k_{i}\bar{x}+b_{i}+e_{i}\\ x_{i},MSC=\frac{x_{i}-b_{i}}{k_{i}} \end{array}$$

where *k_i_* (the regression coefficient) represents the multiplicative scaling factor, *b_i_* (the intercept) denotes the additive baseline drift, and *e_i_* is the residual. In this study, the optimal joint preprocessing strategy was determined by quantitatively evaluating the degree of improvement in the SNR and the sharpness of characteristic peaks afforded by various algorithms. A detailed comparative analysis of the preprocessing performance and spectral feature interpretation is provided in Section 3.1.

### 2.4. Feature Selection and Model Construction

#### 2.4.1. GBDT-Based Nonlinear Feature Wavelength Optimization

Within the 340–1032 nm band, the full-spectrum data exhibit typical characteristics of high dimensionality, high redundancy, and strong multicollinearity. Modeling directly with the full wavelength range is highly susceptible to the introduction of irrelevant background noise and significantly increases the computational overhead.

Given the complex nonlinear mapping relationship between the spectral response and eggshell thickness, this study introduces the GBDT algorithm for feature dimensionality reduction. Unlike traditional linear screening methods such as CARS or Successive Projections Algorithm (SPA), GBDT quantifies the relative contribution of each wavelength variable to the reduction in the loss function during the gradient descent process by constructing an additive regression tree ensemble model based on split gain [28]. For feature *j* (i.e., a specific wavelength point), its global importance *I_j_* is defined as the sum of the squared error gains across all decision trees where that feature serves as a splitting node:

(3) $$\hat{J_{j}^{2}}=\frac{1}{M}\sum_{m=1}^{M}\sum_{t=1}^{L-1}X_{i}\hat{I_{t}^{2}}\cdot ∥(v_{t}=j)$$

where *M* is the number of iterations, *L* represents the number of leaf nodes, *v_t_* denotes the splitting feature of node *t*, and $\hat{I_{t}^{2}}$ represents the reduction in Mean Squared Error (MSE) after splitting node *t*. In this study, variables across the entire spectrum were ranked based on their global feature importance scores. Subsequently, the top 50 feature wavelengths (Top-50) with the highest cumulative contribution were screened as input variables. This strategy effectively eliminated irrelevant spectral information and achieved precise compression of data dimensionality while fully preserving the critical “thickness fingerprint” features.

#### 2.4.2. Improved CatBoost Model Architecture and Anti-Overfitting Strategy

All data processing and modeling in this study were conducted within a Python 3.9 environment. Spectral preprocessing algorithms were implemented using the SciPy v1.17.1 library, while feature optimization and CatBoost modeling were performed using the Scikit-learn (v1.2.2) and CatBoost (v1.2) libraries, respectively. The hardware configuration utilized for the computations consisted of an Intel Core i7-12700H CPU @ 2.30 GHz and 32 GB of RAM.

**Figure 3:**
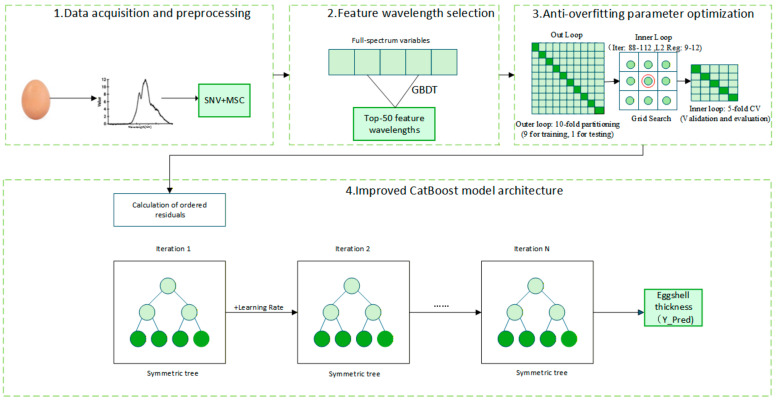
Architecture and optimization strategy of the improved CatBoost model incorporating 10-fold nested cross-validation and bootstrap sampling.

To address the challenges of overfitting and prediction shift—issues frequently encountered by traditional machine learning algorithms when processing small-sample, high-dimensional spectral data—an improved CatBoost regression prediction model was developed. The comprehensive architecture and optimization strategy of this model are illustrated in Figure 3.

The model integrates a robust regression mechanism based on Ordered Boosting and Oblivious Trees. To overcome the gradient estimation bias inherent in traditional GBDT algorithms—which stems from estimating gradients using the entire dataset during iterations—CatBoost calculates residuals by constructing permutations based on “virtual time,” thereby obtaining an unbiased gradient estimate [29]. Simultaneously, the model minimizes an objective function Γ that incorporates a regularization term to enhance its generalization capability:

(4) $$\Gamma (\emptyset )=\sum_{i=1}^{n}L(y_{i},\hat{y_{i}})+\lambda \cdot {∥\omega ∥}_{2}^{2}$$

where *L* denotes the loss function (Root Mean Square Error of Prediction, RMSEP, is employed in this study), and λ represents the L2 regularization coefficient. Furthermore, the model utilizes oblivious trees as base learners, wherein identical splitting features and thresholds are enforced for all nodes at the same level (depth) of the tree. The corresponding decision function can be formulated as follows:

(5) $$f(x)=\sum_{j=1}^{J}c_{j}\cdot ∥(x\in R_{j})$$

This balanced tree structure not only significantly accelerates the model’s prediction speed but, more importantly, effectively mitigates overfitting to local noise, making it exceptionally well-suited for the quantitative analysis of spectral data. To thoroughly eradicate the issue of training set overfitting and accurately assess the model’s generalization performance, this study established a dual anti-overfitting parameter optimization framework integrating nested cross-validation (CV) and Bootstrap sampling.

First, data partitioning was strictly randomized based on independent individual eggs. The model employed a 10-fold nested CV architecture, where 500 independent samples were randomly divided into 10 folds (a 9:1 ratio, yielding 450 samples for calibration and 50 for independent prediction in each iteration). The outer 10-fold loop was utilized to unbiasedly evaluate the model’s generalization capability across various random splits. Concurrently, the inner loop employed a 5-fold CV coupled with a grid search to determine the optimal hyperparameter combination. This inner-outer isolation mechanism, strictly grounded on independent samples, fundamentally eliminated the risks of data leakage and overfitting during parameter tuning.

Second, a Bernoulli distribution-based Bootstrap sampling mechanism [25] was incorporated during model training. The subsampling rate was configured at 0.8, meaning each tree was constructed using only a random 80% subset of the samples. Acting in synergy with robust L2 regularization constraints, this mechanism compelled the model to extract globally robust features rather than memorizing specific data points, thereby successfully minimizing the generalization error on unseen samples.

### 2.5. Model Evaluation Metrics

To quantitatively evaluate the performance of the calibration and prediction models, the *R*^2^ and the *RMSEP* were employed as the fundamental indicators in this study. Furthermore, the residual prediction deviation (*RPD*) was introduced to assess the practical industrial application value of the models. The calculation formulas for these metrics are as follows:

(6) $$R^{2}=1-\frac{\sum_{i=1}^{n}{\left(y_{i}-{\hat{y}}_{i}\right)}^{2}}{\sum_{i=1}^{n}(y_{i}-\bar{y}{)}^{2}}$$

(7) $$RMSEP=\sqrt{\frac{1}{n}\sum_{i=1}^{n}{\left(y_{i}-{\hat{y}}_{i}\right)}^{2}}$$

(8) $$RPD=\frac{SD}{RMSEP}$$

where $y_{i}$ is the actual measured value of the *i*-th sample, ${\hat{y}}_{i}$ is the predicted value, $\bar{y}$ denotes the arithmetic mean of the measured values for all samples, and n represents the total number of samples. *SD* is the standard deviation of the measured values in the prediction set, and *RMSEP* is the root mean square error of prediction. The *RPD* value is interpreted as follows: when RPD < 2.0, the model’s predictive capacity is insufficient for reliable quantitative analysis; when 2.0 ≤ *RPD* < 3.0, the model exhibits good predictive performance and can be utilized for preliminary grading; when *RPD* 3.0, the model demonstrates excellent robustness and high precision, fully satisfying the requirements for industrial online inspection.

## 3. Results

### 3.1. Analysis of Spectral Response Mechanisms and Preprocessing Effectiveness

**Figure 4:**
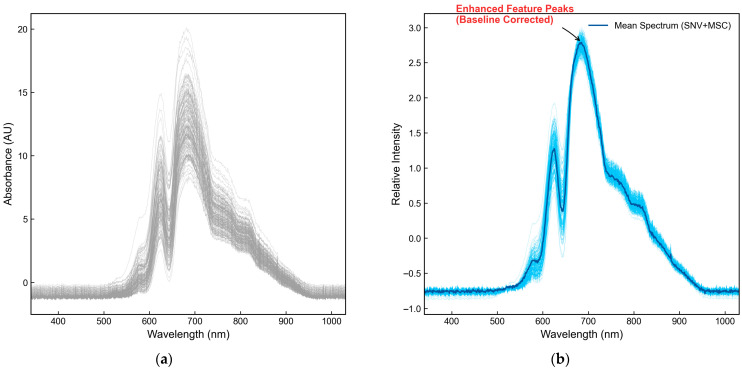
Comparison of spectral characteristics between raw spectra and SNV-MSC preprocessed spectra. (**a**) Raw transmission spectra; (**b**) SNV + MSC preprocessed spectra illustrating baseline correction and enhanced feature peaks.

The raw transmission spectra of the egg samples within the 340–1032 nm range are illustrated in Figure 4a. Observation reveals that the raw spectra exhibit significant baseline drift and scattering noise across the entire wavelength range, primarily due to variations in surface curvature, non-uniform eggshell thickness, and fluctuations in the egg shape index.

Such physical interferences result in a high degree of dispersion among the spectral curves, which severely obscures the subtle chemical fingerprint information associated with the eggshell’s microstructure. As CaCO_3_, the primary constituent of the eggshell, lacks prominent characteristic absorption peaks in the short-wave near-infrared region, the spectral response is predominantly dependent on the overtone vibrations of the internal organic matrix (proteins and membrane lipids) and adsorbed water.

Consequently, eliminating physical optical path differences and enhancing the characteristic signals of the organic matrix are essential prerequisites for achieving robust high-precision modeling.

To identify the optimal signal enhancement strategy, this study conducted a systematic comparison to evaluate the influence of various preprocessing algorithms and their combinations on the model’s predictive performance. The results are summarized in Table 1.

As indicated by the data in Table 1, the baseline model is highly susceptible to scattering noise, yielding relatively low prediction accuracy. Although derivative processing is conventionally employed for baseline correction, it significantly amplified high-frequency noise in this study, deteriorating the model’s performance to levels inferior to those achieved with raw spectra. In contrast, the individual application of either the SNV or MSC algorithm (i.e., omitting the combined step) provided initial corrections for optical path variations and scattering deviations, thereby enhancing model accuracy; however, it remained insufficient to completely eliminate the complex physical interferences.

Further robustness analysis of the preprocessing strategies revealed that the cascade sequence of SNV followed by MSC (SNV + MSC) is crucial for preserving effective features. Reversing this processing order (MSC + SNV) demonstrated that, due to severe individual scattering disparities in the raw spectra, executing the globally mean-dependent MSC first introduces a pseudo-calibration baseline, ultimately leading to a deterioration in RMSEP. Furthermore, prepending a smoothing step to the combined strategy (SG + SNV + MSC) triggered an oversmoothing effect. This excessive smoothing significantly suppressed the subtle yet critical overtone fingerprints of moisture and the organic matrix in the vicinity of 970 nm, resulting in a loss of valuable information and a consequent decline in accuracy. Therefore, prioritizing SNV to eliminate intra-individual multiplicative optical path variations, followed by MSC to unify the global additive baseline, represents the optimal preprocessing trajectory. This synergistic approach maximizes the retention of effective thickness information while achieving superior robustness.

Ultimately, this study employed a joint SNV and MSC cascading strategy to calibrate the raw signals, with the preprocessed spectra illustrated in Figure 4b. It is evident that this strategy effectively eliminated additive baseline offsets and multiplicative scattering errors, leading to a high degree of convergence among the spectral curve clusters and significantly enhancing the resolution of characteristic peaks.

The calibrated spectra exhibit two feature response regions with distinct physical significance. First, the strong absorption region in the visible spectrum at 580–680 nm primarily reflects the deposition of eggshell pigments, such as Protoporphyrin IX. Although the pigments themselves do not constitute thickness, prior research has confirmed that light attenuation within this band maintains a high nonlinear positive correlation with physical eggshell thickness. Second, a weak absorption peak near 970 nm is observed, which is attributed to the second overtone vibration of O-H bonds in water molecules. As noted by Ahmed et al. [14], the biomineralization process of the eggshell is regulated by the organic matrix; thus, variations in water content and protein components serve as critical chemical probes for inverting eggshell compactness and thickness. The significant enhancement of characteristic peaks in Figure 4b indicates that this preprocessing strategy successfully highlighted essential information in these two key bands, establishing a high SNR data foundation for subsequent modeling.

### 3.2. GBDT Feature Wavelength Optimization Results and Physical Significance Analysis

Despite the rich information contained within the full-spectrum data, its high-dimensional variable space—encompassing over 700 dimensions—is characterized by severe multicollinearity and information redundancy. Direct modeling under such conditions is highly susceptible to the introduction of irrelevant noise, which frequently results in overfitting.

**Figure 5:**
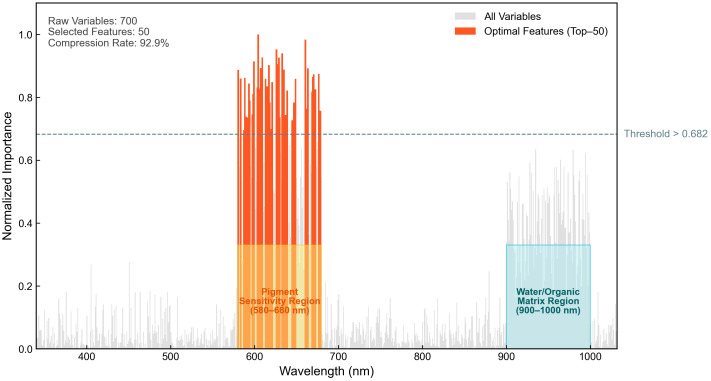
Distribution of GBDT feature importance and identification of physical sensitive regions.

To mitigate these issues, this study utilized the nonlinear feature evaluation mechanism of the GBDT algorithm. Variables across the entire waveband were ranked according to their global importance, quantified by the split gain during the iterative ensemble process. The ranking results of the feature importance are illustrated in Figure 5.

Analysis of the feature distribution reveals that the GBDT algorithm adaptively captured two sensitive regions highly consistent with spectral overtone theory, thereby validating the model’s interpretability. As illustrated by the orange highlighted regions in Figure 5, GBDT assigned the highest feature weights to the 580–680 nm band. This result indicates that for transmission spectral detection, physical light attenuation in the visible region serves as the most direct and sensitive information source for characterizing eggshell thickness, which is perfectly aligned with the aforementioned spectral response mechanism analysis.

Simultaneously, the grey highlighted regions in the figure demonstrate that characteristic variables in the 900–1000 nm band were also extensively retained. This further confirms that the second overtone absorption information of water molecules and the organic matrix near 970 nm plays an irreplaceable role in refining the thickness prediction model. In contrast, low-SNR or non-informative bands located below 400 nm and between 750–850 nm were automatically eliminated by the algorithm.

Ultimately, the number of input variables was drastically compressed from 700 to 50, achieving a feature compression rate of 92.9%. This feature dimensionality reduction strategy—grounded in the dual validation of data-driven results and physical mechanisms—significantly reduces model complexity while fully preserving the most representative physical and chemical features. Consequently, it effectively mitigates the “curse of dimensionality” typically associated with high-dimensional data in small-sample studies.

**Figure 6:**
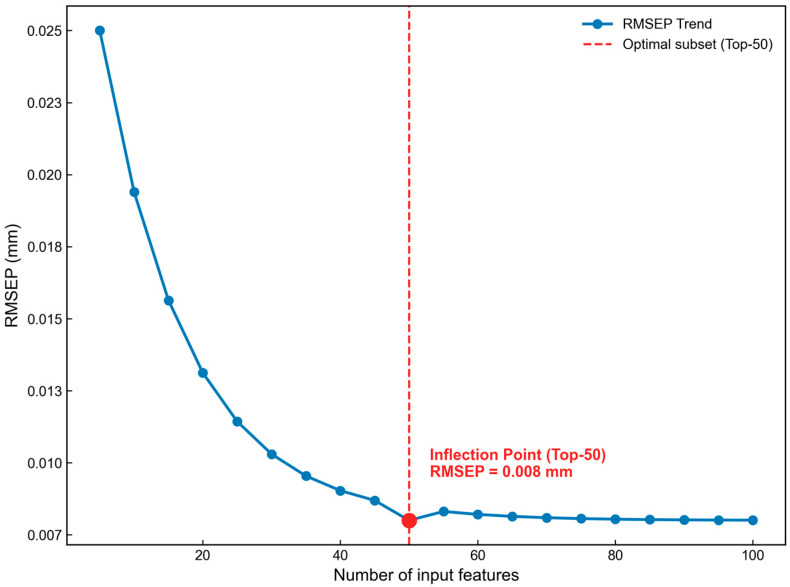
Optimization process of feature number based on GBDT showing the change in RMSEP.

Following the identification of the physical distribution of features, determining the optimal feature subset size is critical to balancing model accuracy and complexity. To this end, this study further analyzed the trend of RMSEP variation as a function of the number of input features, the results of which are illustrated in Figure 6.

As illustrated in Figure 6, with an increasing number of input features, the model error exhibits a sharp initial decline followed by a plateau. When the number of features falls within the 30–40 interval, although the RMSEP rapidly converges to approximately 0.010 mm, the model still fails to adequately incorporate the complete overtone information of the organic matrix and moisture, thus presenting a certain degree of underfitting. As the feature count reaches 50, a distinct “elbow point” emerges on the error curve, corresponding to the minimum RMSEP (0.008 mm). If the feature quantity is further expanded to 60 or beyond, the error not only fails to decrease significantly but instead triggers minor fluctuations in the error curve due to the introduction of redundant background scattering noise, indicating a slight over-tuning phenomenon. In accordance with the principle of parsimony (Occam’s razor), selecting the Top-50 features not only effectively circumvents the risks of both underfitting and over-tuning—strictly governing the critical threshold of generalization capability—but also achieves a 92.9% dimensionality reduction while ensuring maximum prediction accuracy. This constitutes the definitively optimal feature subset from both mathematical and physical mechanistic perspectives.

### 3.3. Predictive Performance and Generalization Robustness of the Improved CatBoost Model

In the eggshell thickness prediction task, the improved CatBoost model constructed with GBDT-selected features demonstrated not only exceptional fitting accuracy but also formidable generalization stability. Across the 10 outer random split iterations, key evaluation metrics exhibited no drastic fluctuations. Specifically, on the prediction set, the model achieved an average coefficient of determination (R^2^_P_) of 0.918 (standard deviation [SD] = 0.005), an RMSEP as low as 0.008 mm (SD = 0.004 mm), and an average RPD of 3.50 (SD = 0.11). Such outstanding and highly consistent performance across different random partitions compellingly demonstrates that the model’s accuracy is not a byproduct of the contingency of a single data split. Furthermore, it firmly validates the efficacy of the Bootstrap sampling strategy in capturing globally robust features.

**Figure 7:**
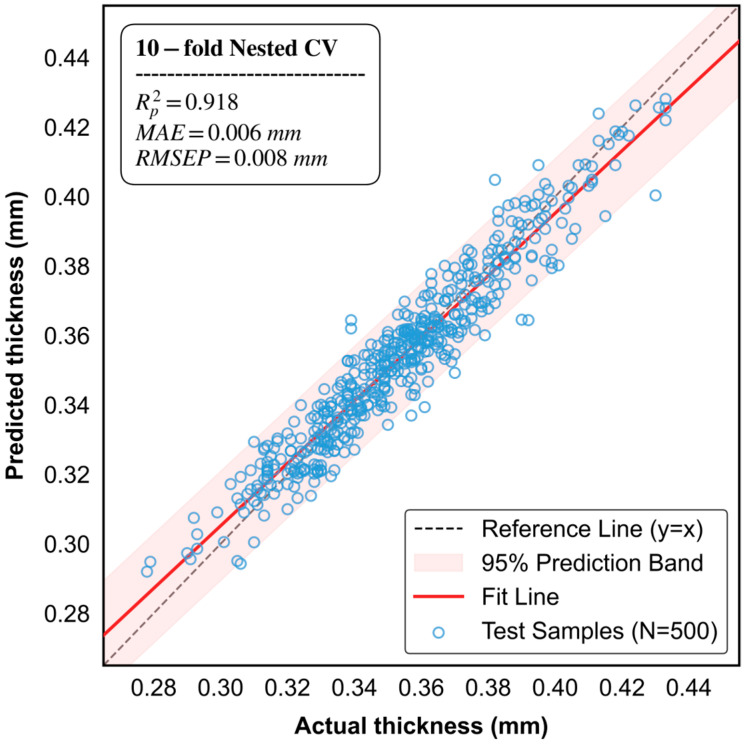
Comparison vs. actual eggshell thickness values for the prediction set using the improved CatBoost model.

As illustrated in Figure 7, the predicted and actual values demonstrate a high degree of consistency, clustering tightly around the 1:1 diagonal line. The 95% prediction interval (represented by the pink shaded area) exhibits a narrow and uniform band distribution across the entire thickness range of 0.280–0.440 mm, suggesting that the model is free from significant nonlinear bias or heteroscedasticity.

This robust predictive performance provides strong evidence for the effectiveness of the Bootstrap resampling strategy in mitigating model overfitting and enhancing the stability of the regression engine.

The exceptional performance of the model is primarily attributed to its unique dual anti-overfitting mechanism. Traditional machine learning models, when processing high-dimensional spectral data, are highly prone to “memorizing” noise within the training set, leading to a situation where R^2^_C_ significantly exceeds R^2^_P_ (a classic symptom of overfitting). To address this, this study implemented a Bootstrap mechanism with a sub-sampling rate of 0.8.

By constructing decision trees based on only 80% of random sub-samples in each iteration, and synergizing this with the oblivious tree splitting criteria inherent to CatBoost, the performance discrepancy between the calibration and prediction sets was successfully constrained within a minimal margin of 0.012.

This confirms that the model has effectively captured the intrinsic physicochemical mapping relationship between eggshell thickness and spectral response, rather than merely performing rote data memorization.

### 3.4. Comparative Analysis of Different Modeling Algorithms

To validate the superiority of the proposed GBDT-CatBoost framework in addressing small-sample and nonlinear spectral modeling tasks, a systematic comparison was conducted against mainstream algorithms prevalent in near-infrared spectroscopy (NIRS) analysis. The comparative models encompass: (1) PLSR, serving as the classical linear regression baseline; (2) SVR, representing traditional nonlinear kernel methods; (3) RF, a representative bagging ensemble algorithm; and (4) XGBoost alongside (5) GBDT, representing two mainstream gradient boosting tree architectures. To ensure an equitable evaluation, all nonlinear models were constructed utilizing the Top-50 characteristic wavelengths selected by GBDT. Furthermore, the hyperparameters of all candidate models were systematically optimized via grid search within the inner loop of the cross-validation. The critical hyperparameter combinations corresponding to the optimal performance of each model (e.g., the penalty coefficient and kernel parameters for SVR, the tree depth for RF) and the comprehensive evaluation metrics are summarized in Table 2.

As demonstrated in Table 2, the PLSR model exhibited suboptimal performance (*R*^2^_P_ = 0.867), indicating that linear models are insufficient to elucidate the complex nonlinear dependencies within eggshell spectra, which are frequently compounded by multiple scattering and organic matrix overtones. In contrast, nonlinear models such as SVR and RF demonstrated improved predictive accuracy; however, the RF model displayed pronounced overfitting, with an *R*^2^_C_ of 0.965 compared to an *R*^2^_P_ of only 0.892, suggesting an over-emphasis on noise details within the training set.

Among the boosting-based ensemble algorithms, while XGBoost and GBDT significantly improved model performance, their generalization capabilities on the small-sample dataset of this study remained inferior to the improved CatBoost model. Specifically, although XGBoost (*R*^2^_P_ = 0.887) and GBDT (*R*^2^_P_ = 0.898) were proficient in modeling complex feature relationships, their performance was constrained by parameter sensitivity when processing high-dimensional spectral data. Conversely, the improved CatBoost algorithm implemented in this study utilized an ordered boosting mechanism, which performs unbiased residual calculations during each gradient estimation step, thereby effectively enhancing the model’s predictive robustness for unseen samples. Consequently, the improved CatBoost model achieved the highest predictive accuracy (RMSEP = 0.008 mm) and the optimal industrial robustness (RPD = 3.50) using a parsimonious set of only 50 input features.

### 3.5. Ablation Study and Mechanism Discussion of Key Improvement Strategies

To further quantify the marginal contributions of the core modules within the proposed improved CatBoost framework—specifically, the GBDT-based feature selection and the Bootstrap resampling mechanism—this study conducted an ablation study based on the control variable method. By monitoring the evolution of model performance throughout the calibration-prediction process, the underlying mechanism for accuracy enhancement was elucidated from the perspective of statistical learning theory. The detailed experimental results are presented in Table 3.

#### 3.5.1. GBDT-Based Feature Sparsification and Multicollinearity Suppression

As presented in Table 3, Strategy I (the baseline model) was constructed directly using the full spectrum (700 variables). Although it achieved superior fitting performance on the calibration set (*R*^2^_C_ = 0.948), its performance on the prediction set significantly deteriorated (*R*^2^_P_ = 0.828), exhibiting pronounced overfitting characteristics. From the perspective of spectral analysis, the full-band data is interspersed with instrumental thermal noise, stray light, and uninformative variables in the edge bands, which constitute “interference background noise” within the high-dimensional space. While minimizing the loss function, CatBoost not only captures valid chemical fingerprints but also erroneously fits these random noises, thereby impairing the model’s generalization capability.

In contrast, with the introduction of the GBDT feature selection module in Strategy II, the model input dimensionality was compressed by 92.9% (to 50 variables). Although *R*^2^_C_ underwent a slight pullback, *R*^2^_P_ leaped to 0.891, and the generalization gap narrowed significantly from 0.120 to 0.051. This qualitative improvement is attributed to the nonlinear information gain mechanism of the GBDT algorithm: it precisely identified and retained characteristic wavebands strongly correlated with the vibrations of the eggshell’s organic matrix (C-H and O-H bonds), such as the 580–680 nm and 970 nm regions, while eliminating redundant variables that induce multicollinearity.

This feature sparsity processing effectively reduced the complexity of the model’s hypothesis space, making it more feasible to approximate the underlying physicochemical mapping laws under small-sample conditions.

#### 3.5.2. Optimization of the Bias-Variance Trade-Off via Bootstrap Resampling

Building upon feature dimensionality reduction, Strategy III further incorporates a Bootstrap mechanism with a sub-sampling rate of 0.8. The experimental data indicate that this strategy successfully compressed the generalization gap to an extreme minimum of 0.012, while concurrently attaining the peak predictive accuracy (RMSEP = 0.008 mm).

The underlying mathematical principle of this enhancement lies in the reconfiguration of the model’s “bias-variance trade-off.” According to ensemble learning theory, the generalization error of a model is fundamentally decomposed into bias, variance, and irreducible noise. Although CatBoost effectively minimizes prediction bias via its ordered boosting mechanism, it remains susceptible to high variance risks—namely, hypersensitivity to training data fluctuations—particularly in the context of small-sample, high-dimensional spectral data.

The Bootstrap mechanism implemented in this study introduces sample-level perturbations during each iteration, artificially diversifying the training distribution. This strategy compels the model to forgo the “rote memorization” of specific outliers and instead extract robust, global spectral patterns. Consequently, this regularization effect, while entailing a marginal sacrifice in training bias (with *R*^2^_C_ adjusting from 0.942 to 0.930), significantly reduces the model’s prediction variance, thereby substantially bolstering its industrial-grade robustness when applied to unseen samples.

**Figure 8:**
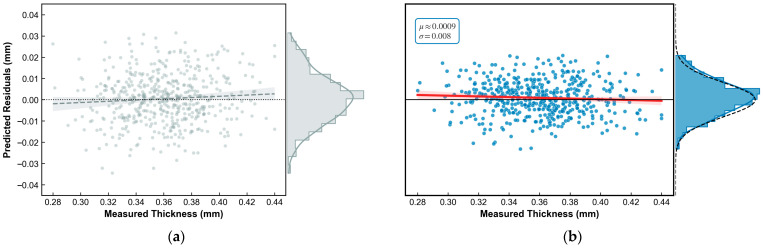
Comparison of prediction residual distributions under different modeling strategies. (**a**) Residual distribution characteristics of the baseline model (Strategy I); (**b**) Residual distribution characteristics of the improved model (Strategy III).

Furthermore, to provide a more intuitive validation of the improved model’s advantages in eliminating systematic errors, the residual distribution characteristics of the baseline model (Strategy I) and the improved model (Strategy III) were comparatively analyzed, as depicted in Figure 8.

As illustrated in Figure 8a, the residual scatter of the baseline model exhibits a certain degree of heteroscedasticity, and the red trend line deviates from the zero axis, indicating that the model suffers from systematic bias when processing samples at the extremes of the thickness range. In contrast, the residuals of the improved model (Figure 8b) follow a near-perfect zero-mean Gaussian distribution (μ≈0, σ = 0.008) across the entire measurement range, with the red trend line being nearly horizontal and coincident with the zero axis. This highly uniform and unbiased residual distribution further confirms that the synergistic strategy of feature optimization and Bootstrap proposed in this study effectively eliminated nonlinear systematic errors in spectral modeling, ensuring consistent reliability of the model across various thickness levels.

## 4. Discussion

This study addressed the challenge of eggshell quality and safety evaluation in the poultry food supply chain by proposing a non-destructive near-infrared spectroscopy (NIRS) detection framework based on GBDT feature selection and an improved CatBoost algorithm. It aims to break through the robustness bottleneck of spectral data parsing algorithms in the non-destructive evaluation of complex food quality. As recent studies indicate, optical non-destructive testing technologies combined with machine learning are becoming a core trend for online quality evaluation in the food industry [30], exhibiting high cross-domain applicability in fruit and vegetable quality identification and meat (e.g., modified atmosphere packaged mutton) freshness prediction [31].

However, the diversity of internal food components, the high-dimensional redundancy of spectral data, and potential interferences in industrial environments often pose severe stability challenges for traditional detection models in practical applications. To overcome this bottleneck, the deep integration of artificial intelligence (AI) tools and advanced feature selection technologies is considered the key [32]. Although previous studies (e.g., Ahmed et al. [14]) have preliminarily confirmed the feasibility of evaluating eggshell thickness using NIRS combined with conventional machine learning, this study achieves significant innovations in both methodological depth and industrial implementation.

Methodologically, traditional models are highly susceptible to falling into local optima or overfitting when processing small-sample, high-dimensional spectra. The GBDT-CatBoost framework constructed in this study adaptively extracted 50 core feature wavelengths highly correlated with the eggshell organic matrix via the GBDT algorithm, effectively stripping away background scattering and redundant information. Simultaneously, it integrated the ordered boosting mechanism with a dual anti-overfitting strategy (10-fold nested cross-validation and Bootstrap resampling) for the first time, fundamentally overcoming the gradient estimation bias of traditional boosting tree algorithms in spectral prediction. This strategy highly aligns with the perspective proposed by Xu et al. [33] that “optimizing feature extraction and enhancing generalization capability are the core to improving the stability of non-destructive testing,” successfully eliminating the systematic bias of residuals in thickness prediction.

In terms of industrial practice, this method maximally compressed the model input dimensions to 50 (a compression rate of up to 92.9%), greatly reducing the computational overhead and model inference latency for online detection. This not only overcomes the time-consuming issue of full-spectrum modeling but also provides a novel algorithmic foundation for the low-cost deployment of high-throughput automated egg grading equipment, laying a solid technical basis for 100% intelligent full inspection of poultry food products on modern production lines.

Despite the ideal results achieved by the proposed GBDT-CatBoost framework under static laboratory conditions, there are still certain limitations regarding its application in actual industrial grading lines that require further exploration in future research. First, the modeling samples in this study were primarily concentrated on a specific breed and shell color. Future research needs to introduce more diverse sample data (e.g., white-shelled, pink-shelled, and eggs with varying depths of shell color) and further investigate the potential temporal evolution effects on the spectral penetration baseline caused by moisture volatilization and air cell enlargement due to storage time (storage age). Second, the online detection environment in industrial settings is complex; high-speed conveying belt vibrations, ambient temperature fluctuations, and dynamic micro-deviations in probe detection distance may all introduce additional nonlinear noise interference. Therefore, future work will focus on collecting multi-batch data under complex industrial conditions and exploring the introduction of domain adaptation or incremental learning mechanisms to continuously enhance the spatiotemporal generalization capability and application robustness of the model in dynamic online detection equipment.

## 5. Conclusions

This study proposed and validated a novel non-destructive detection framework based on near-infrared spectroscopy combined with GBDT dimensionality reduction and an improved CatBoost algorithm, successfully resolving the challenges of high-dimensional spectral redundancy and small-sample overfitting faced in the online evaluation of eggshell thickness. The results demonstrated that eliminating physical scattering interference through a cascade preprocessing of SNV and MSC and utilizing the GBDT algorithm to precisely target core spectral fingerprints related to the eggshell organic matrix and moisture overtones can substantially compress data dimensions without losing critical effective information. Coupled with the CatBoost model incorporating 10-fold nested cross-validation and a Bootstrap sampling mechanism, the framework exhibited outstanding prediction accuracy and cross-sample generalization stability while maintaining extremely low inference latency. This study not only provides an effective solution to the theoretical bottleneck of “balancing feature interpretability and model robustness” in spectral analysis but also establishes a solid technical foundation for realizing low-cost, high-throughput automated non-destructive quality grading in the modern poultry industry.

## Figures and Tables

**Table 1:** Comparison of modeling performance among different spectral preprocessing methods.

Preprocessing Method	*R* ^2^_P_	RMSEP	Performance Analysis
Raw Spectra	0.825	0.015	Baseline model; significantly affected by scattering noise.
SG Smoothing	0.838	0.014	Smooths high-frequency noise; limited improvement on baseline drift.
1st Derivative	0.812	0.016	Eliminates baseline drift but amplifies noise, reducing signal-to-noise ratio.
2nd Derivative	0.795	0.017	Pronounced noise amplification; destroys subtle thickness fingerprint information.
SNV	0.847	0.011	Effectively corrects for light path variations, significantly enhancing accuracy.
MSC	0.890	0.010	Separates additive and multiplicative scattering deviations; outperforms single calibration.
SNV + MSC	0.918	0.008	Synchronous normalization and scattering correction; maximizes preservation of valid information.
MSC + SNV	0.865	0.012	Introduces a pseudo-calibration baseline, disrupting individual correction logic.
SG + SNV + MSC	0.892	0.010	Oversmoothing suppresses the weak but critical overtone signatures in the vicinity of 970 nm.

**Table 2:** Performance comparison and optimal hyperparameters of different modeling methods.

Model	Input Variables	Optimal Parameters	Calibration Set		Prediction Set		RPD
			*R* ^2^_C_	RMSEC	*R* ^2^_P_	RMSEP	
PLSR	Full (700)	n_components = 9	0.885	0.013	0.867	0.015	1.87
SVR	Top-50	kernel = RBF, C = 12.5, γ = 0.05	0.902	0.011	0.881	0.013	2.15
RF	Top-50	n_estimators = 200, max_depth = 8	0.965	0.003	0.892	0.012	2.33
GBDT	Top-50	n_estimators = 250, max_depth = 5, lr = 0.05	0.945	0.005	0.898	0.011	2.80
XGBoost	Top-50	n_estimators = 200, max_depth = 4, lr = 0.05	0.927	0.009	0.887	0.010	2.55
Improved CatBoost	Top-50	depth = 7, L2_reg = 11, iter = 112	0.930	0.006	0.918	0.008	3.50

**Table 3:** Results of the ablation study for key improvement strategies.

Modeling Strategy	Base Algorithm	Input Features	Bootstrap	*R* ^2^_C_	*R* ^2^_P_	Generalization Gap (R^2^_C_—R^2^_P_)
I. Baseline Model	Standard CatBoost	Full spectrum (700)	×	0.948	0.828	0.120
II. Feature Selection	Standard CatBoost	Top-50 (GBDT)	×	0.942	0.891	0.051
III. Proposed Method	Improved CatBoost	Top-50 (GBDT)	×80%	0.930	0.918	0.012

## Data Availability

The original contributions presented in the study are included in the article; further inquiries can be directed to the corresponding author.

## Abbreviations

The following abbreviations are used in this manuscript:

CatBoost	Categorical Boosting
FD	First derivative
GBDT	Gradient Boosting Decision Tree
K	Potassium
MSC	Multiplicative Scatter Correction
N	Nitrogen
NIR	Near-infrared
P	Phosphorus
PLSR	Partial Least Squares Regression
RBF	Radial basis function
RF	Random Forest
RMSEC	Root Mean Square Error of Calibration
RMSEP	Root Mean Square Error of Prediction
RPD	Residual Prediction Deviation
SD	Second derivative
SG	Savitzky–Golay
SNR	Signal-to-noise ratio
SNV	Standard Normal Variate
SPA	Successive Projection Algorithm
SVM	Support Vector Machine
XGBoost	Extreme Gradient Boosting
